# An Antibody of the Secreted Isoform of Disintegrin and Metalloprotease 9 (sADAM9) Inhibits Epithelial–Mesenchymal Transition and Migration of Prostate Cancer Cell Lines

**DOI:** 10.3390/ijms25126646

**Published:** 2024-06-17

**Authors:** Yura Jotatsu, Shain-Ying Sung, Ming-Heng Wu, Shunya Takeda, Yuto Hirata, Koki Maeda, Shiuh-Bin Fang, Kuan-Chou Chen, Katsumi Shigemura

**Affiliations:** 1 Department of Public Health, Kobe University Graduate School of Health Sciences, 7-10-2 Tomogaoka, Suma-ku, Kobe 654-0142, Japan; yura199911.kcao@gmail.com (Y.J.); tyanhira0430@gmail.com (Y.H.); 2 International Ph.D. Program for Translational Medicine, College of Medical Sciences and Technology, Taipei Medical University, Taipei 11031, Taiwanmhwu1015@tmu.edu.tw (M.-H.W.); 3 The Ph.D. Program for Translational Medicine, College of Medical Science and Technology, Taipei Medical University, 250 Wu-Hsing St., Taipei 110, Taiwan; 4 Department of Medical Device Engineering, Kobe University Graduate School of Medicine, 7-5-2 Kusunoki-cho, Chuo-ku, Kobe 650-0017, Japan; shun0906hisha@gmail.com; 5 Department of Urology, Kobe University Graduate School of Medicine, 7-5-2 Kusunoki-cho, Chuo-ku, Kobe 650-0017, Japan; kokimaeda1118@gmail.com; 6 Division of Pediatric Gastroenterology and Hepatology, Department of Pediatrics, Shuang Ho Hospital, Taipei Medical University, 291 Jhong Jheng Road, Jhong Ho District, New Taipei City 23561, Taiwan; sbfang@tmu.edu.tw; 7 Department of Pediatrics, School of Medicine, College of Medicine, Taipei Medical University, Taipei 11031, Taiwan; 8 Department of Urology, Taipei Medical University Shuang Ho Hospital, 291, Zhongzheng Road, Taipei 235, Taiwan; kuanchou@tmu.edu.tw; 9 Department of Urology, Teikyo University School of Medicine, 2-11-1 Kaga, Itabashi-ku, Tokyo 173-8605, Japan

**Keywords:** ADAM9, secreted ADAM9, castration-resistant prostate cancer, invasion, epithelial–mesenchymal transition (EMT)

## Abstract

Prostate cancer (PC) is the most common cancer diagnosed in men worldwide. Currently, castration-resistant prostate cancer (CRPC), which is resistant to androgen deprivation therapy, has a poor prognosis and is a therapeutic problem. We investigated the antitumor effects on PC of an antibody neutralizing secreted disintegrin and metalloproteinase domain-containing protein 9 (sADAM9), which is a blood-soluble form. We performed proliferation assays, wound healing assays, invasion assays, Western blot (WB), and an in vivo study in which a sADAM9 neutralizing antibody was administered intratumorally to PC-bearing mice. In invasion assays, the sADAM9 neutralizing antibody significantly inhibited invasion in all cell lines (TRAMP-C2: *p* = 0.00776, LNCaP: *p* = 0.000914, PC-3: *p* = 0.0327, and DU145: *p* = 0.0254). We examined epithelial–mesenchymal transition (EMT) markers, one of the metastatic mechanisms, in WB and showed downregulation of Slug in TRAMP-C2, LNCaP, and DU145 and upregulation of E-cadherin in TRAMP-C2 and PC-3 by sADAM9 neutralization. In mouse experiments, the sADAM9 neutralizing antibody significantly suppressed tumor growth compared to controls (1.68-fold in TRAMP-C2, 1.89-fold in LNCaP, and 2.67-fold in PC-3). These results suggested that the sADAM9 neutralizing antibody inhibits invasion, migration, and tumor growth in PC. Previous studies examined the anti-tumor effect of knockdown of total ADAM9 or sADAM9, but this study used the new technology of neutralizing antibodies for sADAM9. This may be novel because there was no animal study using a neutralizing antibody for sADAM9 to see the relationship between ADAM9 expression and prostate cancer.

## 1. Introduction

Prostate cancer (PC), the second most frequently diagnosed cancer in men worldwide and the fifth leading cause of cancer death, is expected to continue to increase [1,2]. Risk factors for PC are advanced age, family history of malignancy, and genetic factors [3]. Prostate cancer patients are currently treated with active surveillance, radical prostatectomy, external beam radiation therapy, and endocrine therapy to suppress androgens [4,5,6]. In particular, androgen deprivation therapy is used for metastatic PC, but patients acquire resistance to androgen deprivation therapy in most cases and eventually develop castration-resistant prostate cancer (CRPC), which has a poor prognosis [7,8]. Localized PC has a 5-year survival rate of almost 100%, while metastatic PC, which has mainly metastasized to bones and other organs, has a 5-year survival rate of less than 30% [9,10]. Effective CRPC treatments that can slow or stop metastasis are badly needed.

A disintegrin and metalloproteinase domain-containing protein 9 (ADAM9) is a member of the ADAM family of transmembrane proteins belonging to the zinc protease superfamily. ADAM9 physiologically regulates cell adhesion and intracellular signal transduction in several malignant tumors [11,12,13,14]. In bladder and prostate cancers, knockdown of ADAM9 suppresses cancer cell proliferation [15,16] and inhibits cancer cell migration by suppressing epithelial–mesenchymal transition (EMT), which is related to cancer progression and metastasis [17,18]. ADAM9 expression has been associated with malignancy in bladder, prostate, and renal cancer [15,16,19]. Particularly in PC, ADAM9 promotes the transition from castration-sensitive prostate cancer (CSPC) to CRPC [20].

ADAM9 is structurally composed of a disintegrin domain related to cell adhesion and an extracellular domain (ectodomain), which is a multidomain. Secreted ADAM9 (sADAM9), derived from selective splicing of ADAM9 transcripts [21,22], is also secreted by activated hematopoietic stem cells and promotes colon cancer invasion and liver metastasis [23]. sADAM9 causes migration and metastasis in PC by activating the integrin β1/FAK/AKT signaling pathway. Furthermore, sADAM9 expression is significantly increased in the serum of PC patients, suggesting that sADAM9 may be useful as a serum marker for PC progression [20].

EMT is correlated with carcinogenesis, decreased survival, cancer cell invasion, and metastasis in breast cancer and PC [24,25]. Blocking EMT is one of the key mechanisms for the development of anti-metastatic agents. The purpose of this study is to investigate whether antibody neutralization of sADAM9 has an inhibitory effect on cancer cell growth and EMT and has potential as a new therapeutic agent effective for PC and CRPC.

## 2. Results

### 2.1. Cell Proliferation Assay

**Figure 1:**
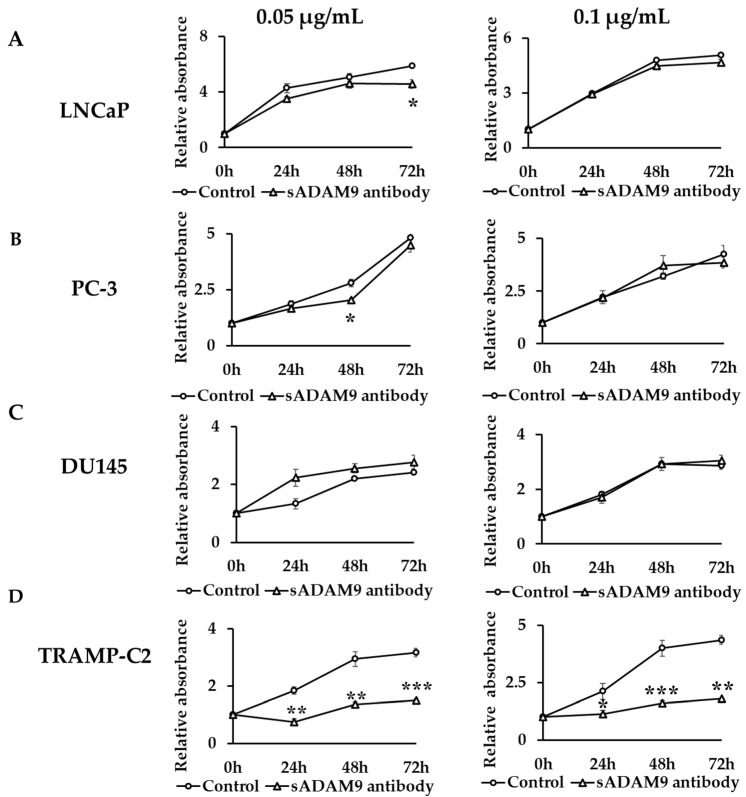
Comparison of cell proliferation change between control groups and sADAM9 neutralizing antibody groups. The absorbance when antibodies were administered in each group was set at 1, and we evaluated the cell proliferation ability by comparing the relative absorbance. (**A**) 0.05 µg/mL sADAM9 neutralizing antibody (sADAM9 antibody) suppressed cell proliferation in LNCaP at 72 h (*p* = 0.0161). (**B**) The PC-3 sADAM9 antibody was suppressed at 48 h (*p* = 0.0131). (**C**) In DU145, the sADAM9 neutralizing antibody did not inhibit cell proliferation significantly. (**D**) In TRAMP-C2, 0.05 µg/mL sADAM9 neutralizing antibody significantly inhibited cell proliferation (24 h: *p* = 0.00343, 48 h: *p* = 0.00411, and 72 h: *p* = 0.000410), and 0.1 µg/mL sADAM9 neutralizing antibody inhibited cell proliferation (24 h: *p* = 0.0473, 48 h: *p* = 0.000681, and 72 h: *p* = 0.00142). *: *p* < 0.05, **: *p* < 0.01, ***: *p* < 0.001.

In LNCaP, a 0.05 µg/mL sADAM9 neutralizing antibody suppressed cell proliferation at 72 h (*p* = 0.0161) (Figure 1A). In PC-3, a 0.05 µg/mL sADAM9 neutralizing antibody suppressed cell proliferation at 48 h (*p* = 0.0131) (Figure 1B). In DU145, the sADAM9 neutralizing antibody did not inhibit cell proliferation significantly (Figure 1C). Furthermore, in TRAMP-C2, 0.05 µg/mL sADAM9 neutralizing antibody significantly inhibited cell proliferation at 24 h (*p* = 0.00343), 48 h (*p* = 0.00411), and 72 h (*p* = 0.000410), and 0.1 µg/mL sADAM9 neutralizing antibody also inhibited cell proliferation at 24 h (*p* = 0.0473), 48 h (*p* = 0.000681), and 72 h (*p* = 0.00142) (Figure 1D).

### 2.2. Wound Healing Assay

**Figure 2:**
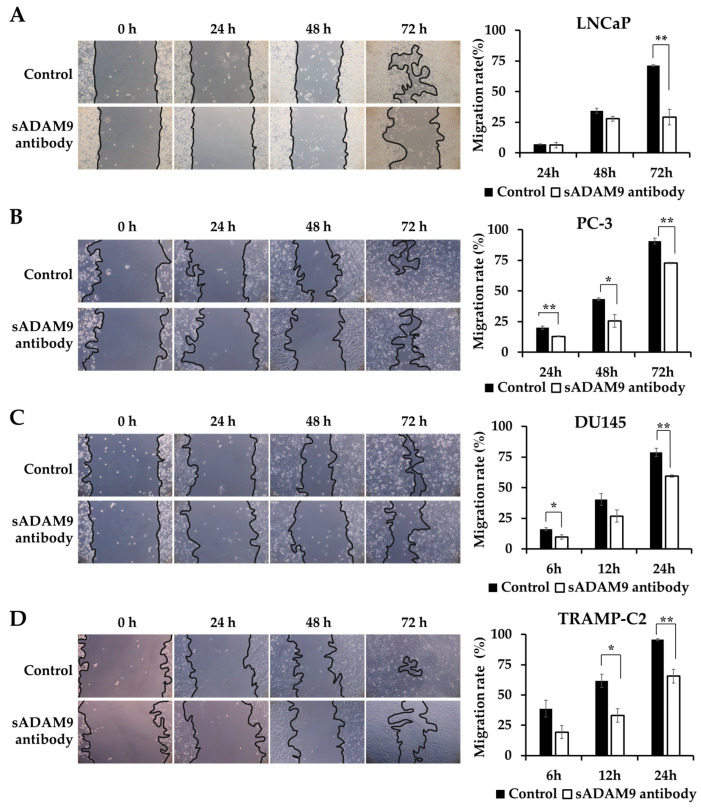
Comparison of the inhibitory effect on wound healing in the sADAM9 neutralizing antibody treatment and control groups. Microscopic images of the changes in the wound and graphs showing the covered area rates calculated from the images are shown. (**A**) The sADAM9 neutralizing antibody (sADAM9 antibody) significantly inhibited wound healing in LNCaP at 72 h (*p* =0.00281). (**B**) The PC-3 antibody was inhibited at 24 h (*p* = 0.00691), 48 h (*p* = 0.0295), and 72 h (*p* = 0.00179). (**C**) In DU145, the sADAM9 neutralizing antibody significantly inhibited wound healing at 6 h (*p* = 0.0410) and 24 h (*p* = 0.00543). (**D**) In TRAMP-C2, the sADAM9 neutralizing antibody significantly inhibited wound healing at 12 h (*p* = 0.0211) and 24 h (*p* = 0.00655) compared to the control. *: *p* < 0.05; **: *p* < 0.01. “Migration rate” means ”rate of covered area” in graphs. These graphs show the rate of wounds that have been closed by cell migration (100× magnification).

We observed the changes in the wounds over time under a microscope and showed the covered area rates calculated from these images as graphs. sADAM9 neutralization significantly inhibited wound healing in LNCaP at 72 h (*p* = 0.00281) (Figure 2A) and in PC-3 at 24 h (*p* = 0.00691), 48 h (*p* = 0.0295), and 72 h (*p* = 0.00179) (Figure 2B). In DU145, the sADAM9 neutralizing antibody significantly inhibited wound healing after 6 h (*p* = 0.0410) and 24 h (*p* = 0.00543) (Figure 2C). In TRAMP-C2, sADAM9 neutralizing antibody treatment also showed significant wound healing inhibition at 12 h (*p* = 0.0211) and 24 h (*p* = 0.00655) (Figure 2D). In addition, we have used human CRPC cell lines (i.e., C4-2B) and demonstrated significant inhibition by sADAM9 neutralization treatments (Supplementary Figure S1).

### 2.3. Cell Invasion Assays

**Figure 3:**
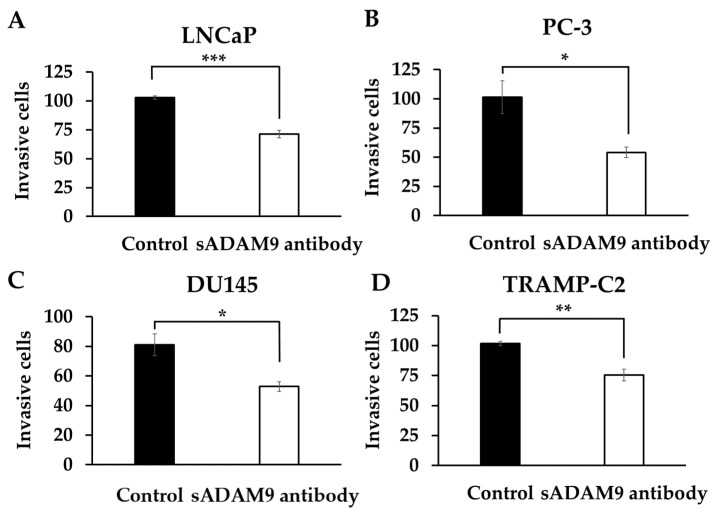
Comparison of the inhibitory effect of the sADAM9 neutralizing antibody on invasive ability in treated and control groups. sADAM9 neutralizing antibody (sADAM9 antibody) decreased invasive cells in LNCaP (**A**), PC-3 (**B**), DU145 (**C**), and TRAMP-C2 (**D**) (LNCaP: *p* = 0.000914, PC-3: *p* = 0.0327, DU145: *p* = 0.0254, and TRAMP-C2: *p* = 0.00776). *: *p* < 0.05, **: *p* < 0.01, ***: *p* < 0.001.

In LNCaP, the sADAM9 neutralizing antibody reduced invasive cells compared to controls (*p* = 0.000914) (Figure 3A). In PC-3 (Figure 3B), DU145 (Figure 3C), and TRAMP-C2 (Figure 3D), the sADAM9 neutralizing antibody groups also had fewer invasive cells than the control groups (PC-3: *p* = 0.0327, DU145: *p* = 0.0254, and TRAMP-C2: *p* = 0.00776).

### 2.4. Western Blot (WB) Analysis

**Figure 4:**
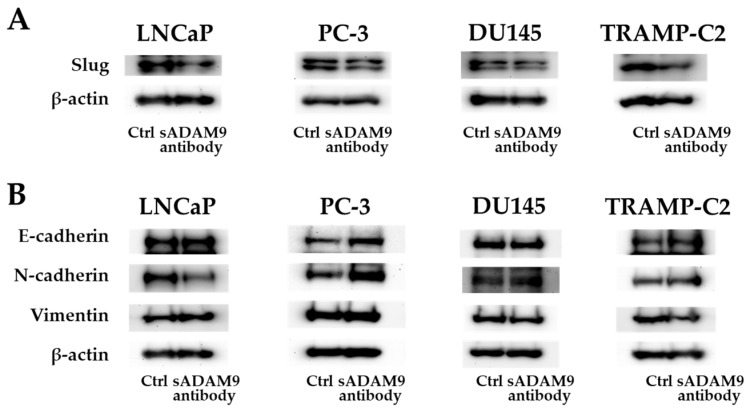
Protein expression levels of E-cadherin, N-cadherin, Vimentin, and Slug. (**A**) The sADAM9 neutralizing antibody decreased the expression of Slug, an EMT induction marker, in LNCaP, DU145, and TRAMP-C2. (**B**) The sADAM9 neutralizing antibody (sADAM9 antibody) increased the protein expression levels of the epithelial marker E-cadherin compared to the control (Ctrl) group in TRAMP-C2 and PC-3 (**B**). The expression of N-cadherin, a mesenchymal marker, decreased in LNCaP, and Vimentin decreased in DU145 after sADAM9 neutralization. The molecular weights of E-cadherin, N-cadherin, Vimentin, Slug, and beta-actin were 120 kDa, 99.7 kDa, 54 kDa, 30 kDa, and 42 kDa, respectively.

As for Slug, the sADAM9 neutralizing antibody decreased the expression in LNCaP, DU145, and TRANP-C2, but in PC-3, there was no significant difference between the control and sADAM9 neutralizing antibody groups (Figure 4A). In LNCaP, the expression of N-cadherin decreased with the administration of the sADAM9 neutralizing antibody (sADAM9 antibody) (Figure 4B). The sADAM9 neutralizing antibody increased the expression of E-cadherin in PC-3 (Figure 4B) and decreased the expression of Vimentin in DU145 (Figure 4B). Furthermore, in TRAMP-C2, the sADAM9 neutralizing antibody increased the expression of E-cadherin and decreased the expression of Vimentin compared to the control (Ctrl) group (Figure 4B).

### 2.5. Animal Experiments

**Figure 5:**
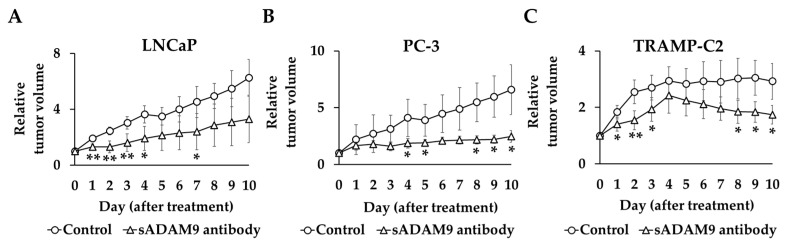
Relative tumor volume changes in sADAM9 neutralizing antibody and control groups in mice prostate cancer models. We set the tumor volume before each antibody administration as 1 and compared the relative tumor volumes of the control and treated groups. (**A**) The sADAM9 neutralizing antibody significantly inhibited tumor growth in LNCaP (Day 1: *p* = 0.00274, Day 2: *p* = 0.00276, Day 3: *p* = 0.00711, Day 4: *p* = 0.0140, and Day 7: *p* = 0.0382). (**B**) In PC-3, antibodies inhibited tumor growth (Day 4: *p* = 0.0500, Day 5: *p* = 0.0460, Day 8: *p* = 0.0417, Day 9: *p* = 0.0356, and Day 10: *p* = 0.0450). (**C**) In TRAMP-C2, the sADAM9 neutralizing antibody also significantly inhibited tumor growth (Day 1: *p* = 0.0394, Day 2: *p* = 0.00619, Day 3: *p* = 0.0343, Day 8: *p* = 0.0280, Day 9: *p* = 0.0136, and Day 10: *p* = 0.0165). *: *p* < 0.05; **: *p* < 0.01.

The tumor volume before administration of the antibody in each group was set at 1, and the relative tumor volume was compared between the treatment and control groups (Figure 5). In TRAMP-C2, the sADAM9 neutralizing antibody significantly inhibited tumor growth on day 1 (*p* = 0.0394), day 2 (*p* = 0.00619), day 3 (*p* = 0.0343), day 8 (*p* = 0.0280), day 9 (*p* = 0.0136), and day 10 (*p* = 0.0165). The relative tumor volumes in LNCaP were also significantly smaller in the sADAM9 neutralizing antibody group than controls on day 1 (*p* = 0.00274), day 2 (*p* = 0.00276), day 3 (*p* = 0.00711), day 4 (*p* = 0.0140), and day 7 (*p* = 0.0382). The sADAM9 neutralizing antibody inhibited tumor growth in PC-3 on day 4 (*p* = 0.0500), day 5 (*p* = 0.0460), day 8 (*p* = 0.0417), day 9 (*p* = 0.0356), and day 10 (*p* = 0.0450).

## 3. Discussion

Progression to CRPC is a fatal condition that significantly reduces the survival rate in advanced PC. Currently, androgen deprivation therapy is the standard of care for advanced PC [4,5]. Clarification of the risk factors for CRPC progression and the development of novel molecularly targeted agents for CRPC could improve the survival rate for CRPC.

We focused on sADAM9, a blood-soluble form of ADAM9 related to PC progression. The sADAM9 neutralizing antibody significantly suppressed cell proliferation in both CSPC and CRPC cell lines. The sADAM9 neutralizing antibody also significantly suppressed tumor growth in mice transplanted with TRAMP-C2, LNCaP, and PC-3. ADAM9 knockdown also inhibited tumor growth in mice with subcutaneously implanted PC cells [26]. No previous animal studies have focused on the function of sADAM9.

Regarding the sADAM9 neutralizing effect in inhibiting migration and invasion, we conducted the wound healing assay and invasion assay. From these assays, the sADAM9 neutralizing antibody significantly reduced the cell migratory and invasive abilities of all PC cells used in this study.

EMT is a phenomenon in which epithelial cells are converted into motile mesenchymal cells [27], and we investigated EMT as the main phenomenon causing cell migration and invasion. Slug is one of the EMT inducers; E-cadherin is a marker of epithelial cells; and N-cadherin and Vimentin are markers of mesenchymal cells [28,29]. When EMT is suppressed, the expression of E-cadherin increases while that of Slug, N-cadherin, and Vimentin decreases.

We observed changes in the protein-level expression of these markers in cells treated with a sADAM9 neutralizing antibody. In TRAMP-C2, sADAM9 neutralization increased the expression of E-cadherin and decreased Slug and Vimentin, indicating an inverse phenotype for EMT. The expression of Slug was also decreased in LNCaP and DU145 by sADAM9 neutralizing antibody treatment, indicating that EMT was suppressed in these cells as well. The sADAM9 neutralizing antibody also decreased N-cadherin in LNCaP and Vimentin in DU145. Since sADAM9 promoted EMT and pathological progression in PC in a previous study [24], a therapeutic strategy using the sADAM9 antibody to inhibit EMT might slow PC progression and improve survival. In fact, regarding the neutralization effect of the antibody towards sADAM9, we have periodically observed the expressions of sADAM9 (0, 5, 30, and 60 min later on antibody exposure) by Western blots in those cell lines of TRAMP, LNCaP, PC3, and DU145 but only saw a significant change in DU145. This demonstrated such data; these findings (ADAM9 in DU145) were supported by our previous study using conditioned medium in DU145 [11].

Cell proliferation assays were conducted by seeding cells on flat-bottomed plates in vitro to examine the cell proliferation ability in two dimensions. The in vivo study examined cell proliferation in three dimensions by observing tumor growth in mice subcutaneously implanted with PC. In cell proliferation assays, the sADAM9 neutralizing antibody significantly inhibited TRAMP-C2 and temporarily inhibited cell proliferation in human cell lines. However, in vivo, the sADAM9 neutralizing antibody significantly reduced tumor growth compared to controls throughout the experiment. We suggest that sADAM9 inhibited tumor growth more comprehensively in vivo than in vitro because the sADAM9 neutralizing antibody also inhibited invasion and migration, as shown above (Figure 2 and Figure 3). The significant tumor-inhibiting effect of sADAM9 in vivo may thus be a cumulative effect.

As to the molecular mechanism of cancer inhibition, ADAM9 activated EGFR by a precursor of shutting down Heparin-Binding Epidermal Growth Factor-like Growth Factor (pro HB-EGF), then producing HB-EGF, a ligand of soluble EGFR, then binding to the EGFR-downstream-intracellular signal-transduction cascade, including the AKT pathway for activation, then promoting normal and tumor cell growths [11]. In addition, those integrins that interact with ADAM9 are α2β1, α6β1, α6β4, α9β1, and αVβ5, and such interactions induce migration of fibroblasts and cancer cell invasion by cell-migration activation [11]. As this paper shows, ADAM9 relates to EMT, suggesting ADAM9 inhibition leads to a stop to cancer migration, invasion, and metastasis [11]. Our in vivo data, not in vitro ones, of cancer inhibition demonstrated that the 3D condition, not the 2D condition, was reflected by those factors of cancer development such as EMT, so this means ADAM9 inhibition of EMT could decrease cancer expansion, resulting in cancer inhibition. In addition, as to the FAK/AKT signaling pathway, we have demonstrated sADAM9-inducing migration and metastasis of prostate cancer via the FAK/AKT signaling pathway in our previous study [20], and Chen et al. also showed the RAB11A protein played a pivotal role in cell malignant progression and tumor formation of prostate cancer via this signaling pathway [30].

Our study has several limitations. First, the number of mice was small, and the duration of the animal study was limited. Second, we only explored the mechanism of the anti-tumor effect of the sADAM9 neutralizing antibody within the EMT pathway. Third, we have missed the model of TRAMP-C2 inhibition in castrated mice in an in vivo study. Fourth, we have missed the animal study to see tumor burden with human CRPC cells such as C4-2 and 22Rv1 in castrated mice. Fifth, our observation time during the sADAM9 antibody in animal studies as well as in vitro studies may be short. Sixth, our incubation time with TGF-beta may be long in the wound healing assay. In future studies, we will explore other pathways that may be involved in the metastasis-inhibiting effects of the sADAM9 neutralizing antibody.

In conclusion, the administration of the sADAM9 neutralizing antibody suppressed tumor growth, migration, and invasion both in vitro and in vivo and inhibited EMT in most PC cells. Based on these results, the sADAM9 neutralizing antibody may have potential as a novel PC treatment.

## 4. Materials and Methods

### 4.1. Cell Lines and Cell Culture

We used LNCaP, PC-3, and DU145 human cell lines and TRAMP-C2 cell lines derived from mice. LNCaP is a CSPC cell line, and C4-2B, PC-3, DU145, and TRAMP-C2 are CRPC cell lines. RPMI-1640 medium (with 10% fetal bovine serum and 1% penicillin-streptomycin) was used for LNCaP, PC-3, and DU145, and Dulbecco’s Modified Eagle’s Medium (D-MEM) (high glucose) (with 10% fetal bovine serum and 1% penicillin-streptomycin) was used to culture TRAMP-C2. All cell line cultures were conducted in a 5% CO_2_ humidified atmosphere at 37 °C.

### 4.2. Reagents and Chemicals

We obtained Human/Mouse ADAM9 Ectodomain Antibody (Cat# AF949) and Normal Goat IgG Control (Cat#AB-108-C) from R&D Systems, Minneapolis, MN, USA.

### 4.3. Proliferation Assays

The effects of the sADAM9 neutralizing antibody on cell proliferation in LNCaP, PC-3, DU145, and TRAMP-C2 cells were measured by MTS (3-(4,5-dimethylthiazol-2-yl)-5-(3-carboxymethoxyphenyl)-2-(4-sulfophenyl)-2H-tetrazolium) (Promega, Madison, WI, USA) assays. We seeded LNCaP, PC-3, DU145, and TRAMP-C2 cells on 96-well plates at 2.0 × 10^3^ cells/well (n = 3) for two-dimensional cell culture under conditions of 37 °C, 5% CO_2_, for 24 h. Then, 0.1 µg/mL sADAM9 neutralizing antibody and normal goat IgG at the same concentration were added, and the absorbance over time (490 nm) was measured for 72 h.

### 4.4. Wound Healing Assays

Wound healing assays were used to evaluate the invasion and migration abilities of cells. All cells were seeded on 12-well plates at 1.0 × 10^5^ cells/mL (n = 3) and cultured for 24 h. After 24 h, we added 5 ng/mL transforming growth factor-β to induce EMT. Then, after making wounds with the tips, we washed cells with PBS, added 0.1 µg/mL sADAM9 neutralizing antibody and 0.1 µg/mL control goat IgG, and cultured cells for 24 h. After the addition of these reagents, DU145 and TRAMP-C2 cells were observed for 24 h, LNCaP and PC-3 cells for 72 h, and C4-2B cells for 96 h using the EOS utility (Canon, Tokyo, Japan). The results were analyzed using ImageJ (Java, MD, USA).

### 4.5. Cell Invasion Assay

Invasion analysis was performed to evaluate cell invasive potentials using the CytoSelect 24-well Cell Invasion Assay kit (Cat# CBA-110, Cell Biolabs Inc., San Diego, CA, USA). LNCaP, PC-3, DU145, and TRAMP-C2 cells were prepared in serum-free medium at 1.0 × 10^6^ cells/mL each, and 0.1 µg/mL sADAM9 neutralizing antibody and goat IgG were added to 24 well plates (n = 3). Moreover, 48 h later, three views of each sample were randomly selected, and invasive cells were counted by microscope.

### 4.6. Western Blot (WB) Analysis

WB was performed to examine the expression of the EMT markers E-cadherin, N-cadherin, and Vimentin and the EMT inducer Slug at the protein level to determine the mechanism of action of the sADAM9 neutralizing antibody. All cells were seeded in 6-well plates at 1 ×10^5^ cells/well. After 24 h, 0.5 µL/mL of TGF-β was added, and after another 24 h, 0.1 µg/mL of sADAM9 neutralizing antibody and goat IgG were added and cultured for 48 h. In this study, anti-E-cadherin (Cosmo bio, Tokyo, Japan), anti-N-cadherin (Proteintech Group Inc., Rosemont, IL, USA), anti-Vimentin (Proteintech), anti-Slug (Cell Signaling Technology, Danvers, MA, USA), and anti-β-actin (Santa Cruz Biotechnology, Dallas, TX, USA) were used. We used HRP-conjugated secondary antibodies (anti-IgG (H+L chain) (mouse) pAb-HRP or anti-IgG (H+L chain) (rabbit) pAb-HRP (MBL, Tokyo, Japan) as secondary antibodies.

### 4.7. Animal Experiments

Animal experiments were performed using LNCaP, PC-3, and TRAMP-C2 mouse models to investigate the antitumor effects of the sADAM9 neutralizing antibody in vivo. PC cells (1.5 × 10^6^ cells/mouse) and Matrigel were mixed and transplanted into 7-week-old C57BL/6 and Balb/c nu-nu mice (LNCaP and TRAMP-C2: n = 5, PC-3 (control group): n = 4, and PC-3 (sADAM9 neutralizing antibody group): n = 5). After the long diameter of the tumor exceeded 7 mm, these mice were divided into a control group (0.1 µg/g goat IgG) and a treatment group (0.1 µg/g sADAM9 neutralizing antibody). These reagents were administered intratumorally. Tumor diameter was measured daily, and relative tumor volume was calculated as (long diameter) × (short diameter)^2^ × 0.5. Mouse body weights were also measured for safety evaluation.

### 4.8. Ethical Approval

All experimental plans and procedures were reviewed and approved by the Institutional Ethics Committee and the Animal Welfare Committee of Kobe University (approval number: P231101).

### 4.9. Statistical Analysis

Comparisons between control and treatment groups were conducted using the Student’s *t*-test. Statistical differences between means were considered significant at *p* < 0.05.

## 5. Conclusions

The administration of the sADAM9 neutralizing antibody suppressed tumor growth, migration, and invasion both in vitro and in vivo and inhibited EMT in most PC cells. Based on these results, the sADAM9 neutralizing antibody may have potential as a novel PC treatment.

## Data Availability

The raw data supporting the conclusions of this article will be made available by the authors upon request.

## Supplementary Materials

The following supporting information can be downloaded at: https://www.mdpi.com/article/10.3390/ijms25126646/s1.
