# Supplementary material for: An Antibody of the Secreted Isoform of Disintegrin and Metalloprotease 9 (sADAM9) Inhibits Epithelial–Mesenchymal Transition and Migration of Prostate Cancer Cell Lines
Source: Int J Mol Sci. 2024 Jun 17;25(12):6646. doi: 10.3390/ijms25126646 (PMC11203924; doi:10.3390/ijms25126646)
Supplement: Supplementary file 1 [file ijms-25-06646-s001.zip › ijms-2929831-supplementary.pdf]

Supplementary Figure S1

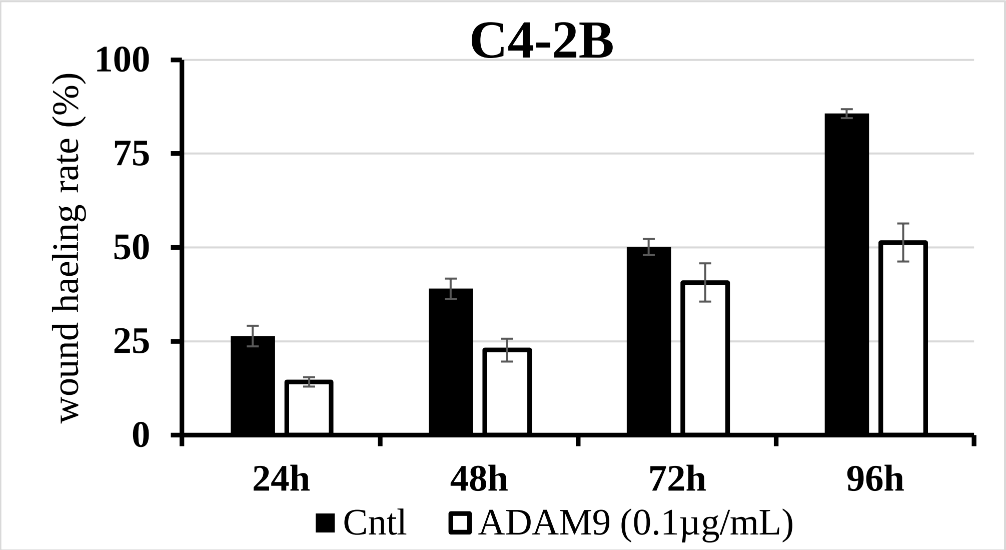

Comparison of the inhibitory effect on wound healing in the sADAM9 neutralizing antibody treatment and control groups. Microscopic images of the changes in the wound and graphs showing the covered area rates calculated from the images are shown. (A) sADAM9 neutralizing antibody (sADAM9 antibody) significantly inhibited wound healing in C4-2B at 24, 48, 72 and 96h ($p < 0.05$).
